# Handedness and individual roll-angle specialism when plunge diving in the northern gannet

**DOI:** 10.1098/rsbl.2023.0287

**Published:** 2023-09-06

**Authors:** Ashley Bennison, Bethany L. Clark, Stephen C. Votier, John L. Quinn, Jamie Darby, Mark Jessopp

**Affiliations:** ^1^ British Antarctic Survey, Madingley, Cambridge CB3 0ET, UK; ^2^ BirdLife International, Cambridge CB2 3QZ, UK; ^3^ Lyell Centre, Heriot-Watt University, Edinburgh EH14 4BA, UK; ^4^ School of Biological, Earth and Environmental Sciences (BEES), University College Cork, Cork T23 N73K, Ireland; ^5^ MaREI, Centre for Marine & Renewable Energy, Environmental Research Institute, University College Cork, Cork T23 N73K, Ireland

**Keywords:** handednesses, specialisms, lateralization, repeatability, gannet, seabird

## Abstract

Many vertebrates show lateralized behaviour, or handedness, where an individual preferentially uses one side of the body more than the other. This is generally thought to be caused by brain lateralization and allows functional specializations such as sight, locomotion, and decision-making among other things. We deployed accelerometers on 51 northern gannets, *Morus bassanus*, to test for behavioural lateralization during plunge dives. When plunge diving, gannets ‘roll’ to one side, and standard indices indicated that 51% of individuals were left-sided, 43% right-sided, and 6% ‘non-lateralized’. Lateralization indices provide no measure of error and do not account for environmental covariance, so we conducted two repeatability analyses on individuals' dive roll direction and angle. Dive side lateralization was highly repeatable among individuals over time at the population level (*R* = 0.878, *p* < 0.001). Furthermore, roll angle was also highly repeatable in individuals (*R* = 0.751, *p* < 0.001) even after controlling for lateralized state. Gannets show individual specializations in two different parts of the plunge diving process when attempting to catch prey. This is the first demonstration of lateralization during prey capture in a foraging seabird. It is also one of the few demonstrations of behavioural lateralization in a mixed model approach, providing a structure for further exploring behavioural lateralization.

## 1. Introduction

Behavioural lateralization, or handedness, is the process of preferentially using one side of the body over the other. This arises from functional brain asymmetry, and may be due to both genetics and environment [1]. Lateralization is widespread among vertebrates including fish [2], amphibians [3], reptiles [4], birds [5] and mammals [6]. Different hemispheres of the brain are thought to control different activities, thereby influencing lateralization [7], which likely evolved as a mechanism to avoid costly duplication of neural circuitry for the same or similar functions [8].

The strength and direction of laterality can vary greatly [9], and many species that show laterality also show a population bias towards one direction [10]. Many lateralized behaviours are tied to survival, including predator vigilance and escape response [11]. For example, Australian magpie, *Cracticus tibicen*, individuals favoured their left eye when making anti-predator alarm calls [12]. Lateralization can also allow animals to engage in multiple behaviours simultaneously such as predator avoidance and food processing; Rogers *et al*. [13] found that domestic chickens, *Gallus gallus domesticus* that were more lateralized in their behaviour were better at discerning food pellets on mixed substrate while also remaining vigilant for predators.

Typically, behavioural lateralization is measured using lateralization indices [14] which use repeated measures of behaviour and record which body side the behaviour occurred on. The ratio of these events can be used to provide a representative lateralization index. Indices have been used to determine lateralization in species including humans [15], killer whales *Orcinus orca* [16], and birds of prey [17]. However, observed lateralization could be caused by poor experimental design or by temporary environmental effects that change any given behaviour (such as weather [18], time of day [19], or location [20]), rather than intrinsic and consistent among-individual differences over time [21,22]. Repeatability analyses are now widely used in behavioural studies [23,24] and address this by estimating, on a scale of 0–1 (from no to perfect repeatability), how individuals behaviourally differ from one another, over two or more independent sample periods. Repeatability is estimated using random variance components in a mixed model framework, in which known confounding effects can also be added as fixed or random effects. This allows for a much more statistically robust approach to understanding behaviour and, importantly, how it may be impacted by multiple causes or cues. Although repeatability analyses are now widespread in the animal behaviour literature, few attempts have been made to estimate repeatability in the context of lateralization [22,25,26].

Lateralization can be theoretically expressed in any species, although it is more apparent in species that engage in demanding or complicated tasks such as prey handling or detection [8,27]. Seabirds provide a good example of this, as they must locate and capture highly mobile and ephemeral prey. Though penguins have been shown to engage in lateralized behaviour during aggressive encounters [28,29] and Caspian terns, *Hydroprogne caspia*, are documented handling prey in a lateralized manner [30], no information exists on the occurrence of lateralization during seabird foraging. The northern gannet, *Morus bassanus*, hereafter gannet, is a long-lived seabird with a complex foraging behaviour that includes visual detection of prey when in flight [31] followed by plunge diving to a depth of 1.6–14.9 m [32] while simultaneously avoiding conspecific collisions. At the start of a plunge dive, gannets roll to one side, presumably maintaining visual contact with sighted prey. Lateralization of rolling behaviour during plunge dives could potentially help gannets to engage in specialist behaviour that requires multiple actions (e.g. diving, prey visualization, and collision avoidance) to occur at the same time [33]. Here we use gannet-borne accelerometers to (i) estimate the level of lateralization in dive direction during diving using the standard lateralization index; (ii) estimate the repeatability of dive direction using a repeatability framework; and (iii) after controlling for lateralization, examine whether individuals differ from one another in dive roll angle.

## 2. Methods

Research was approved by the University College Cork Animal Ethics Committee, the University of Exeter Ethics Committee, and conducted under licence from the National Parks and Wildlife Service, Natural Resources Wales, and the British Trust for Ornithology.

### (a) Data collection

Breeding adult gannets attending 3–4 week-old chicks were tracked from Great Saltee, Ireland (52^o^ 7′ 37.92″, −6^o^ 35′ 45.6″) and Grassholm, Wales, UK (51° 43′ N, 05° 28′ W). Birds were caught using an 8–10 m pole with a metal crook, weighed, and equipped with a combination of dataloggers. Birds were tagged for an average of 3.25 ± 1.62 days and were equipped with: GPS loggers (i-gotU GT-120, Mobile Action Technology Inc., Taipei, Taiwan, 14 g); time depth recorders (TDR, CEFAS G5, 2.5 g); and tri-axial accelerometers (Gulf Coast Data Concepts X16-mini, 17 g) recording G-forces (1 g = 9.807 m s^2^) at 50 Hz. GPS and TDR loggers were attached ventrally to 2–3 central tail feathers using strips of waterproof tape. Accelerometers were attached to 10–15 mantle feathers between the wings to ensure proximity to the centre of gravity required for accurate accelerometer readings [34] using strips of waterproof tape. Fourteen accelerometer-equipped birds were tracked in 2015, 20 in 2016, 31 in 2017, and six in 2018. Total instrument mass was less than 2% of body mass and positioned to minimize both aerodynamic and hydrodynamic drag [35]. It was not possible to undertake post-tagging monitoring due to the limited nature of fieldwork. However, all birds were seen attending to chicks prior to and following tag removal. Whilst it is not possible to entirely rule out any effect of tag attachment, the limited weight deployed on individual birds, combined with appropriate tag placement and chick attendance indicates a strong confidence that behaviour and breeding success was not compromised. For birds from Great Saltee, 2–3 breast feathers were plucked for genetic sexing following the method outlined by Griffiths *et al.* [36].

### (b) Data processing and behaviour classification

Plunge diving gannets roll to one side, fold back their wings, and then plunge at the ocean surface [31]. Dives were identified from accelerometry using threshold analysis [37,38]. Dives occurred when average acceleration (running average of 2 s) in the x-axis was less than 0 g and standard deviation in the mean x-axis was greater than 1.4 g following Bennison *et al.* [39]. Roll was calculated as the rotation of the individual in the x-axis and is calculated using the following formula:

$$\mathrm{Roll}=\left(\frac{y}{\sqrt{x^{2}+\;z^{2}}}\right)\;\times \;\left(\frac{180}{\pi }\right)$$

where *x* is the acceleration in the forward-facing surge channel, *y* is the acceleration in the lateral sway channel, and *z* represents the acceleration in the vertical heave channel. Roll is calculated from −180° to +180°, where negative values are left-sided roll and positive values a right-sided roll from a horizontal position. Roll was calculated every second and we used the mean roll in the 5 s preceding a dive based on field observations to determine the length of time that would sufficiently capture a roll behaviour. Field observations were undertaken during preceding field observations and during time spent sea watching.

**Figure 1.**
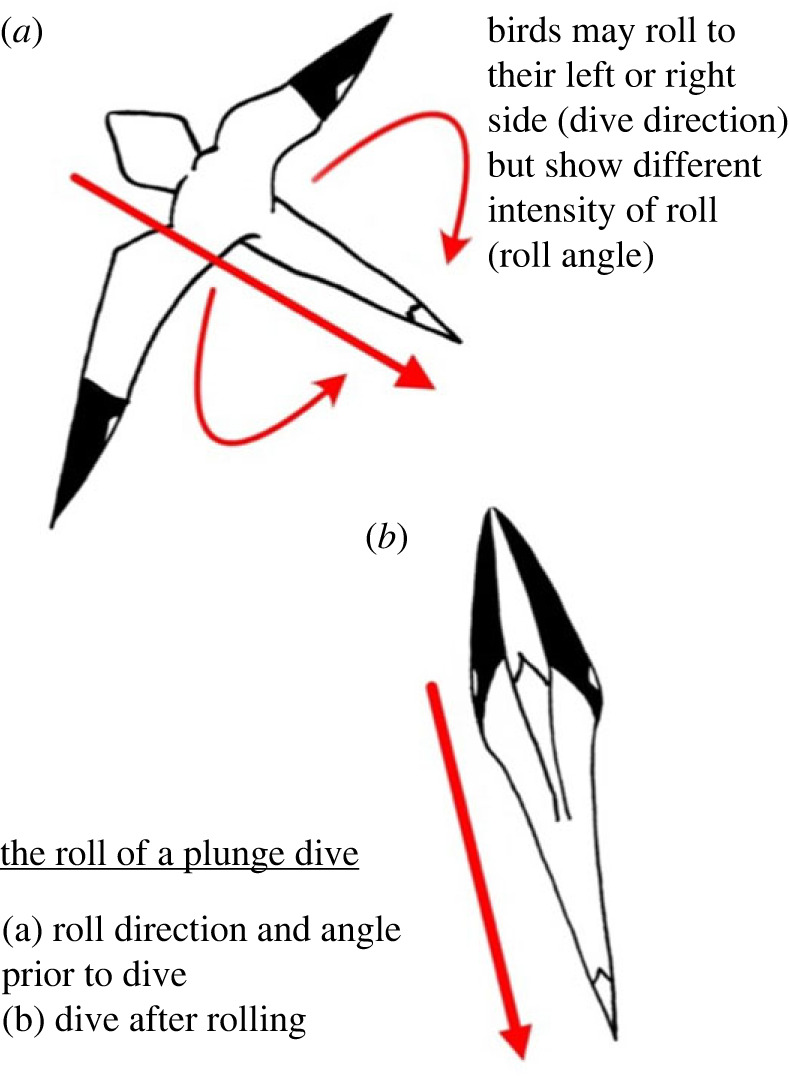
The roll associated with the plunge dive of a gannet can be considered as either roll direction (to the left or right side of the bird's body) or as the angle of roll from a horizontal position.

Roll was recorded as both roll direction and roll angle (figure 1). When gannets plunge dive and roll, this is recorded in degrees from 0. A ‘dive direction’ is recorded from a dive to the birds left or right side of the body, and intensity to which the bird rolls is recorded as ‘roll angle’ and is degrees from 0.

### (c) Pre-dive lateralization

Pre-dive lateralization analysis was undertaken using the lateralization index (LI) from [14]:

$$\mathrm{LI}=\frac{Rd-Ld}{Rd+Ld}$$

where *Rd* is equal to the number of right roll dives and *Ld* is equal to left roll dives. LI ranges from −1 to 1; where −1 shows 100% of dive rolls to be left-sided, whilst 1 is 100% of dive rolls to be right-sided. The LI proportions determined whether individuals have a right-side bias (LI = 1.0 to 0.25), a left side bias (LI = −1 to −0.25), or no bias (LI = −0.25 to 0.25) as initially proposed in key studies of lateralization [10,40,41]. A Wilcoxon rank sum test tested for sex-specific lateralization in the birds from both colonies.

It is possible that any distribution of left/right lateralized animals may be an artefact of sample size. To determine if the observed distribution held enough statistical power to match the wider population, a post-hoc power analysis was undertaken. The ‘pwr’ package in R [42] assessed whether the distribution of lateralized individuals found in this study was representative of the wider population where confidence/power was set to *α* > 0.80; representing an 80% probability that the documented distribution was representative of the wider population in the result of a significant *p* value. This analysis was only undertaken on lateralized individuals to determine if the ratio between left- and right-sided birds was consistent across a population.

### (d) Repeatability of pre-dive roll direction and angle

We estimated the individual repeatability of dive direction (1/0 = left/right dive) using the rptR package in R [23], with gannet ID and the date of dive (day) as random effects. Date was included to account for local environmental conditions such as weather systems or sea state (see discussion for further information on local environmental conditions). We also estimated the repeatability of roll angle with gannet ID and date as random effects. This model also included a bird's lateralized state (left-handed, right-handed, neutral) to determine how lateralized state accounts for differences in dive roll. Models were run using a bootstrapping approach, where model outcome was compared to 1000 randomized permutations of the same data set to simulate a null data set. Mean R-value models were then compared using a likelihood ratio test. Dive direction models used a binomial data structure, dive angle models used a Gaussian data structure. The rptR package estimates repeatability for the random terms specified, and the higher the value of R, the more repeatable (consistently different) individuals are from one another. In the context of individual handedness, the random effect ‘gannet ID’ provides an estimate of how repeatable individual differences are within our population of gannets for (i) roll direction and (ii) roll angle.

## 3. Results

### (a) Pre-dive lateralization

**Figure 2.**
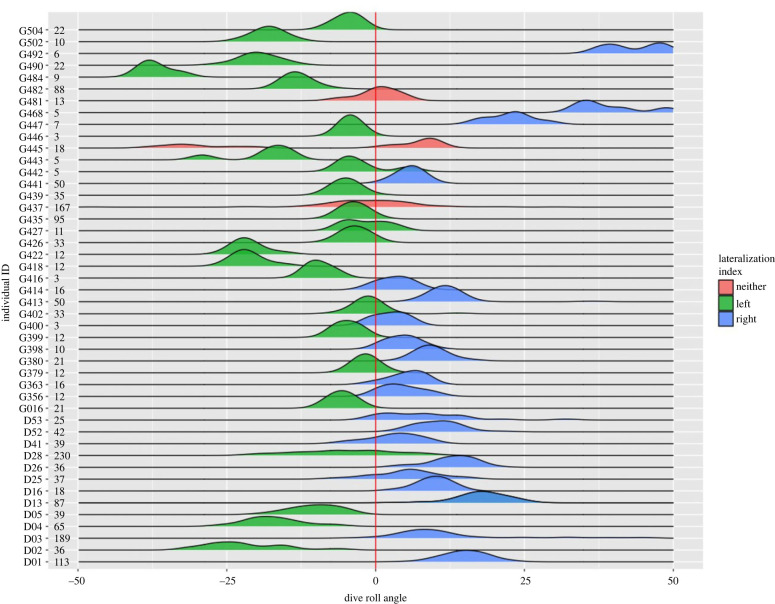
Distribution of roll in pre-dive behaviour for gannets tagged with accelerometers. The red line crosses through 0 where non-lateralized individuals would be expected to be distributed. Gannet ID is labelled on the left axes followed by number of dives recorded, with density distribution shown for each animal. Distributions are coloured to represent the designation of ‘left’, ‘right’, or ‘neither’ as described by the LI. Distributions are presented as geom_density functions form the ggplot2 and ggridge packages in R [43,44]. These are kernel density estimates and can be considered as smoothed versions of histograms and are designed to provide a non-binned density distribution of continuous data and have scales relevant to the number of dives for each bird.

From 71 tagged gannets, accelerometry identified dives for 14 birds from Great Saltee and 37 from Grassholm, successfully recording 2133 dives. LI scores were consistent with lateralized behaviour for 48 birds (LI scores < −0.25 or > 0.25 for all individuals), 22 right-sided birds and 26 left-sided birds, with three neutral birds (figure 2; electronic supplementary material, table S1–S4). There was no difference in lateralization among the sexes when testing between the Great Saltee birds (five females/eight males Wilcoxon rank sum test: *W* = 198 *p* = 0.65).

Power analysis suggested the described distribution of right/left lateralized birds is likely to be representative of the true distribution, even if the *p* value only tends towards the *p* < 0.05 threshold (*h* = −1.00, *n* = 48, α > 0.80, *p* = 0.0623).

### (b) Repeatability analyses of dive direction and angle

Individuals showed strong repeatability of roll direction (*R* = 0.878 ± 0.0512, *p* < 0.001, *n* = 2130, 51 birds). The repeatability estimate associated with date was low (*R* = 0.037 ± 0.024, *p* < 0.001) suggesting little to no effect of environmental variability at the scale of day. The bootstrapped repeatability estimates provide an average R value of 0.878 (CI 0.739–0.944).

Roll angle was also highly repeatable (*R* = 0.751 ± 0.0537, *p* < 0.001, 2130 observations from 51 birds) and the repeatability dropped slightly when accounting for the lateralization state of left/right/neither (*R* = 0.749 ± 0.0572, *p* < 0.001, 2130 observations from 51 birds). Date repeatability was higher for roll angle than in the roll direction model (*R* = 0.141 ± 0.0487, *p* < 0.001).

## 4. Discussion

Northern gannets exhibited behavioural lateralization while rolling prior to plunge dives, with an approximately even split between left- and right-sided birds. To our knowledge, this is the first study demonstrating behavioural lateralization of a seabird species during prey capture attempts. Lateralized prey capture implies a link to sensory lateralization; the hemispheric processing of prey information [16], in this case potentially through visual cues. As gannets are visual predators which locate prey before a dive [45], lateralization during prey capture likely increases successful foraging, though the mechanism behind this is unknown. Visual lateralization in birds can affect vigilance [46], navigation [47] and prey discrimination [48]. It is possible that gannets gain cognitive benefits from engaging in lateralized behaviour such as plunge diving. As animals specialize behaviours on one side of the body to minimize neural development cost [8], this may allow for hemispheric asymmetries which can provide advantages for specific behaviours. Brain regions have previously been associated behavioural traits [49] and previous research suggests that right-biased birds have better prey discrimination and prey handling [50], whilst left-biased birds may benefit from heightened predator detection and aggression [12,51].

Studies of lateralization use indices to demonstrate individuals differ in their lateralization. However, these indices do not account for the fact that behavioural traits might be affected by environmental conditions [21,22]. Thus, we used repeatability analyses to show that individuals repeatably rolled to the left or right (*R* = 0.87) in a mixed model analysis. Lateralized state drives the direction of a dive roll, but we found that lateralization alone did not drive the repeatability of roll angle (a drop in R from 0.751 to 0.749). This shows individuals differed consistently in the magnitude of the roll angle after controlling for lateralization. This suggests that in addition to lateralization, an additional mechanism drives roll angle specialization. Consistent differences among individuals within a population and behavioural diversification can often be the cause of animals exploiting different niches in an ecosystem [52] and this may explain why gannets show differences in dive roll angle. However, further work is required to fully understand the individual level effects of roll on prey capture in gannets.

At a population level, animals may be lateralized to one direction or another [9] with a lateralized population bias thought to evolve through coordinating behaviour with other asymmetrical conspecifics [53]. Gannets show a lack of population bias in lateralized behaviour reflecting the specialization in individual behaviour rather than conspecific coordination. Lateralized dives may ensure that visual observation of prey is possible during the complicated process of plunge diving. An alternative is that the roll may help individuals enter the water in the safest or most appropriate fashion to facilitate successful prey capture, though the exact process remains unknown. Further exploration of this could be achieved either by the deployment of further tagging devices (such as video equipment) or higher resolution GPS to investigate if there are further fine-scale behaviours that were not possible to detect in the current study. Further observation of gannets foraging at sea would enable documentation of other events such as the presence of conspecifics, other seabirds, and environmental conditions such as weather in relation to lateralized dives.

Lateralized behaviours can be influenced by environmental factors. Seabirds use wind to facilitate movement [54], and it is possible that lateralized behaviour may be affected by wind. A pilot analysis paired lateralized data with GPS tracking and wind data to determine if lateralization was associated with rolling into or out of headwinds; while neither appeared to be the case, the coarse scale of data meant it was not possible to discern whether individuals were reorientating themselves prior to dives (A Bennison & M Jessopp 2019, unpublished data). It is therefore possible that lateralized gannet dives may have further interactions with wind. Repeatability estimates remained significant after controlling for day effects, which absorb effects like wind, and were relatively small yet significant. Different weather conditions may require gannets to modify behaviour to increase success during prey capture. Further research may consider environmental factors such as wind, using high-resolution GPS and wind data.

Lateralization is widely prevalent amongst animal species, and understanding how it ties with wider animal behaviour may reveal how behavioural processes can inform a species' ecology [7]. It is not known how behavioural lateralization may affect larger patterns of behaviour in northern gannets, and how this may be reflected at the population level. The benefits of lateralization are poorly understood in seabird foraging, and this emerging field may provide important context for behavioural and ecological studies.

## Data Availability

Data have been made available at all stages, and are provided with publication. Data also available from the Dryad Digital Repository: https://doi.org/10.5061/dryad.s1rn8pkdh [55]. Further data are provided in the electronic supplementary material [56].
